# Complete genome sequence of *Methanocorpusculum labreanum* type strain Z

**DOI:** 10.4056/sigs.35575

**Published:** 2009-09-24

**Authors:** Iain J. Anderson, Magdalena Sieprawska-Lupa, Eugene Goltsman, Alla Lapidus, Alex Copeland, Tijana Glavina Del Rio, Hope Tice, Eileen Dalin, Kerrie Barry, Sam Pitluck, Loren Hauser, Miriam Land, Susan Lucas, Paul Richardson, William B. Whitman, Nikos C. Kyrpides

**Affiliations:** 1 Joint Genome Institute, 2800 Mitchell Drive, Walnut Creek, CA 94598; 2 Microbiology Department, University of Georgia, Athens, GA 30602; 3 Oak Ridge National Laboratory, Oak Ridge, TN 37830

**Keywords:** archaea, methanogen, Methanomicrobiales

## Abstract

*Methanocorpusculum labreanum* is a methanogen belonging to the order Methanomicrobiales within the archaeal kingdom Euryarchaeota. The type strain Z was isolated from surface sediments of Tar Pit Lake in the La Brea Tar Pits in Los Angeles, California. *M. labreanum* is of phylogenetic interest because at the time the sequencing project began only one genome had previously been sequenced from the order Methanomicrobiales. We report here the complete genome sequence of *M. labreanum* type strain Z and its annotation. This is part of a 2006 Joint Genome Institute Community Sequencing Program project to sequence genomes of diverse Archaea.

## Introduction

*Methanocorpusculum labreanum* is a methanogen belonging to the order Methanomicrobiales within the archaeal kingdom Euryarchaeota. Strain Z is the type strain of this species. It was isolated from surface sediments of Tar Pit Lake at the La Brea Tar Pits in Los Angeles [1]. Most of the other described members of this family have been isolated from anaerobic digesters or waste water [2]. The genus covers organisms with a wide temperature range. One psychrotolerant strain was isolated from a Russian pond polluted with paper mill waste water [3], while other strains were found in heated sediment at a hydrothermal vent site [4]. *Methanocorpusculum* species may be common in subsurface environments as they were the most prominent genus found in a coal bed in Indiana [5] and in shale in northern Michigan [6].

**Figure 1:**
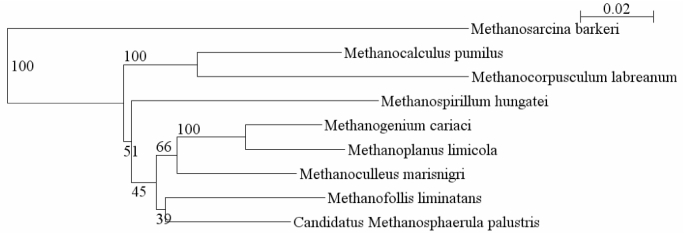
Phylogenetic tree of 16S rRNA of selected Methanomicrobiales showing the distance between the three organisms for which complete genomes are available – *Methanospirillum hungatei*, *Methanocorpusculum labreanum*, and *Methanoculleus marisnigri*. The tree uses sequences aligned within the Ribosomal Database Project (RDP), and the tree was constructed with the RDP Tree Builder [8]. *Methanosarcina barkeri* was used as the outgroup. The numbers indicate bootstrap values based on 100 replicates.

Methanogens have been divided into two groups known as Class I and Class II based on phylogeny [7]. Class I includes the orders Methanococcales, Methanobacteriales, and Methanopyrales, which use H_2_/CO_2_ or formate as substrates for methanogenesis, although some can also use alcohols as electron donors. Class II includes the orders Methanosarcinales and Methanomicrobiales. Some of the Methanosarcinales are capable of using various methyl compounds as substrates for methanogenesis including acetate, methylamines, and methanol, but Methanomicrobiales are restricted to the same substrates as the Class I methanogens [2]. Therefore, Methanomicrobiales are phylogenetically closer to Methanosarcinales but physiologically more similar to Class I methanogens, making them an interesting target for genome sequencing. In a 2006 Community Sequencing Program (CSP) project, we proposed sequencing two members of the order Methanomicrobiales: *M. labreanum* and *Methanoculleus marisnigri*. Previously only one genome was available from this order, that of *Methanospirillum hungatei*. *Methanocorpusculum labreanum* and *Methanoculleus marisnigri* are phylogenetically distant from each other and from *Methanospirillum hungatei* (Figure 1), and they represent the three families within the order Methanomicrobiales. We report here the sequence and annotation of *M. labreanum* type strain Z.

## Organism information

*Methanocorpusculum labreanum* Z was isolated from surface sediments at the La Brea Tar Pits [1]. A polypropylene bottle was filled with half surface sediment and half lake water. In an anaerobic chamber the contents of the bottle were mixed to suspend the sediment, and 0.5 ml of the slurry was added to 5 ml enrichment medium. The enrichment medium contained sodium formate, trypticase peptone, and salts. The gas phase was H_2_-CO_2_ at a ratio of 4:1 and a pressure of 152 kPa. The physiological characteristics of *M. labreanum* were described as follows [1]. The cells were coccoid with a diameter of 0.4-2.0 μm. They were irregular in shape under some growth conditions, such as higher salt or with added acetate. Motility was not observed and no flagella were observed. Growth was observed on H_2_/CO_2_ or formate, but not with acetate, propionate, methanol, trimethylamine, or ethanol. Growth was observed in a narrow window of pH, from 6.5 to 7.5, with pH 7.0 as the optimal value. Growth was observed between 25 and 40°C, with an optimum at 37°C. *M. labreanum* can tolerate a wide range of salt concentration, from 0 to 30 g/L NaCl. Acetate was stimulatory at lower salt concentrations. Either trypticase peptone, yeast extract, or cysteine was required for growth. The features of *M. labreanum* Z are presented in Table 1.

**Table 1:** Classification and general features of *Methanocorpusculum labreanum* Z in accordance with the Minimum Information about a Genome Sequence (MIGS) recommendations [9].

**MIGS ID**	**Property**	**Term**	**Evidence Code**
	Current classification	Domain *Archaea*	
		Phylum *Euryarchaeota*	
		Class *Methanomicrobia*	
		Order *Methanomicrobiales*	
		Family *Methanocorpusculaceae*	
		Genus *Methanocorpusculum*	TAS [10]
		Species *Methanocorpusculum labreanum*	TAS [1]
	Gram stain	negative	TAS [1]
	Cell shape	irregular coccus	TAS [1]
	Motility	nonmotile	TAS [1]
	Sporulation	nonsporulating	
	Temperature range	25-40°C	TAS [1]
	Optimum temperature	37°C	TAS [1]
MIGS-6.3	Salinity	0-30 g/L NaCl	TAS [1]
MIGS-22	Oxygen requirement	anaerobe	TAS [1]
	Carbon source	CO_2_, acetate	TAS [1]
	Energy source	H_2_/CO_2_, formate	TAS [1]
MIGS-6	Habitat	sediment	TAS [1]
MIGS-15	Biotic relationship	free-living	TAS [1]
MIGS-14	Pathogenicity	none	
	Biosafety level	1	
	Isolation	sediment	TAS [1]
MIGS-4	Geographic location	Tar Pit Lake, La Brea Tar Pits	TAS [1]
MIGS-5	Isolation time	1989	TAS [1]
MIGS-4.1 MIGS-4.2	Latitude-longitude	34.107811/-118.599658	
MIGS-4.3	Depth	0-5 cm	TAS [1]
MIGS-4.4	Altitude	not applicable	

Evidence codes - IDA: Inferred from Direct Assay (first time in publication); TAS: Traceable Author Statement (i.e., a direct report exists in the literature); NAS: Non-traceable Author Statement (i.e., not directly observed for the living, isolated sample, but based on a generally accepted property for the species, or anecdotal evidence). These evidence codes are from the Gene Ontology project [10]. If the evidence code is IDA, then the property should have been directly observed, for the purpose of this specific publication, for a live isolate by one of the authors, or an expert or reputable institution mentioned in the acknowledgements.

## Genome sequencing information

### Genome project history

*M. labreanum* was selected for sequencing based upon its phylogenetic position relative to other methanogens of the order Methanomicrobiales. It is part of a 2006 Joint Genome Institute Community Sequencing Program project that included six diverse archaeal genomes. A summary of the project information is shown in Table 2. The complete genome sequence was finished in January, 2007. The GenBank accession number for the project is CP000559. The genome project is listed in the Genomes OnLine Database (GOLD) [11] as project Gc00506. Sequencing was carried out at the Joint Genome Institute (JGI) Production Genomics Facility (PGF). Quality assurance was done by JGI-Stanford. Finishing was done at JGI-PGF. Annotation was done by JGI-Oak Ridge National Laboratory (ORNL) and by JGI-PGF.

**Table 2:** Genome sequencing project information

**MIGS ID**	**Characteristic**	**Details**
MIGS-28	Libraries used	3kb, 6kb and 40kb (fosmid)
MIGS-29	Sequencing platform	ABI3730, 454
MIGS-31.2	Sequencing coverage	34x
MIGS-31	Finishing quality	Finished
	Sequencing quality	less than one error per 50kb
MIGS-30	Assembler	Newbler, Paracel
MIGS-32	Gene calling method	CRITICA, Glimmer
	GenBank ID	CP000559
	GenBank date of release	February 2, 2007
	GOLD ID	Gc00506
	NCBI project ID	18109
	IMG Taxon ID	640069317
MIGS-13	Source material identifier	DSM 4855
	Project relevance	Tree of Life

### DNA isolation, genome sequencing and assembly

The methods for DNA isolation, genome sequencing and assembly for this genome have previously been published [12].

### Genome annotation

Protein-coding genes were identified using a combination of CRITICA [13] and Glimmer [14] followed by a round of manual curation using the JGI GenePRIMP pipeline [15]. GenePRIMP points out cases where gene start sites may be incorrect based on alignment with homologous proteins. It also highlights genes that appear to be broken into two or more pieces, due to a premature stop codon or frameshift, and genes that are disrupted by transposable elements. All of these types of broken and interrupted genes are labeled as pseudogenes. Genes that may have been missed by the gene calling programs are also identified in intergenic regions. The predicted CDSs were translated and used to search the National Center for Biotechnology Information (NCBI) nonredundant database, UniProt, TIGRFam, Pfam, PRIAM, KEGG, COG, and InterPro databases. Signal peptides were identified with SignalP [16], and transmembrane helices were determined with TMHMM [17]. CRISPR elements were identified with the CRISPR Recognition Tool (CRT) [18]. Paralogs are hits of a protein against another protein within the same genome with an e-value of 10^-2^ or lower. The tRNAScanSE tool [19] was used to find tRNA genes. Additional gene prediction analysis and manual functional annotation was performed within the Integrated Microbial Genomes Expert Review (IMG-ER) platform [20].

### Genome properties

**Figure 2:**
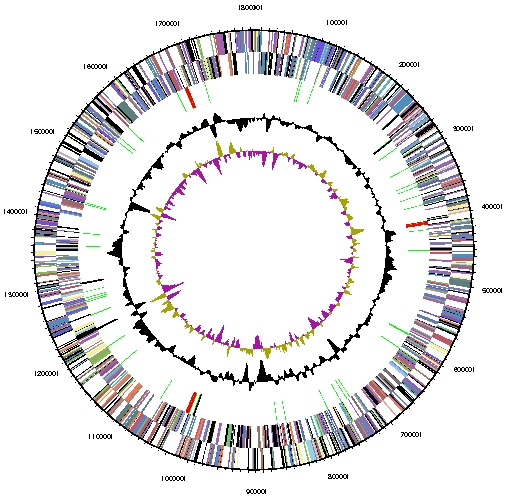
Graphical circular map of the chromosome of Methanocorpusculum labreanum Z. From outside to the center: Genes on forward strand (colored by COG categories), Genes on reverse strand (colored by COG categories), RNA genes (tRNAs green, rRNAs red, other RNAs black), GC content, GC skew.

The genome of *M. labreanum* Z consists of a single circular chromosome (Figure 2). The genome size of 1.80 Mbp is similar to those of Class I methanogens, but smaller than the genomes of *Methanosarcina* species and the other Methanomicrobiales, which range between 2.5 and 5.8 Mbp. The G+C percentage is 50.0%, higher than that of most other sequenced methanogens. There are 1,830 genes, of which 1,765 are protein-coding genes and the remaining 65 are RNA genes. There were only 26 pseudogenes identified, constituting 1.4% of the total genes. The properties and statistics of the genome are summarized in Table 3, and genes belonging to COG functional categories are listed in Table 4.

**Table 3:** Genome statistics

**Attribute**	**Value**	**% of total**
Genome size (bp)	1,804,962	100.00%
DNA coding region (bp)	1,600,673	88.68%
DNA G+C content (bp)	902,600	50.01%
Number of replicons	1	
Extrachromosomal elements	0	
Total genes	1830	100.00%
RNA genes	65	3.55%
rRNA operons	3	
Protein-coding genes	1765	96.45%
Pseudogenes	26	1.42%
Genes in paralog clusters	745	42.21%
Genes assigned to COGs	1358	76.94%
Genes assigned Pfam domains	1335	75.64%
Genes with signal peptides	406	23.00%
Genes with transmembrane helices	368	20.85%
CRISPR repeats	2	

**Table 4:** Numbers of genes associated with the 25 general COG functional categories.

**Code**	**value**	**% of total**	**COG category**
E	130	7.4	Amino acid transport and metabolism
G	54	3.1	Carbohydrate transport and metabolism
D	10	0.6	Cell cycle control, cell division, chromosome partitioning
N	5	0.3	Cell motility
M	35	2.0	Cell wall/membrane/envelope biogenesis
B	2	0.1	Chromatin structure and dynamics
H	129	7.3	Coenzyme transport and metabolism
Z	0	0.0	Cytoskeleton
V	13	0.7	Defense mechanisms
C	134	7.6	Energy production and conversion
W	0	0.0	Extracellular structures
S	172	9.7	Function unknown
R	219	12.4	General function prediction only
P	95	5.4	Inorganic ion transport and metabolism
U	17	1.0	Intracellular trafficking, secretion, and vesicular transport
I	24	1.4	Lipid transport and metabolism
Y	0	0.0	Nuclear structure
F	49	2.8	Nucleotide transport and metabolism
O	57	3.2	Posttranslational modification, protein turnover, chaperones
A	0	0.0	RNA processing and modification
L	65	3.7	Replication, recombination and repair
Q	8	0.5	Secondary metabolites biosynthesis, transport and catabolism
T	30	1.7	Signal transduction mechanisms
K	77	4.4	Transcription
J	147	8.3	Translation, ribosomal structure and biogenesis
-	293	16.6	Not in COGs

## Insights from the genome sequence

The genome sequence of *M. labreanum* Z shows some similarities to Class I methanogens and some to Methanosarcinales but also has some unique features. In common with Class I methanogens, *M. labreanum* uses a partial reductive TCA cycle to synthesize 2-oxoglutarate, and it has the Eha membrane-bound hydrogenase. Similar to Methanosarcinales, *M. labreanum* has the Ech membrane-bound hydrogenase. A unique feature of *M. labreanum* and the other Methanomicrobiales is the presence of anti- and anti-anti-sigma factors, which is surprising as Archaea do not use sigma factors. Phylogenetic analysis of methanogenesis and cofactor biosynthesis enzymes suggest that Methanomicrobiales form a group distinct from other methanogens, and therefore methanogens can be split in to three classes [12]. Surprisingly *M. labreanum* lacks the F_420_-nonreducing hydrogenase, which has been proposed to couple Coenzyme M-Coenzyme B heterodisulfide reduction and ferredoxin reduction for the first step of methanogenesis in the cytoplasm of Methanomicrobiales [21]. In place of this hydrogenase, *M. labreanum* may use the membrane-bound hydrogenase Mbh or energy-converting hydrogenase Ech to couple heterodisulfide reduction to a transmembrane ion gradient [12].
